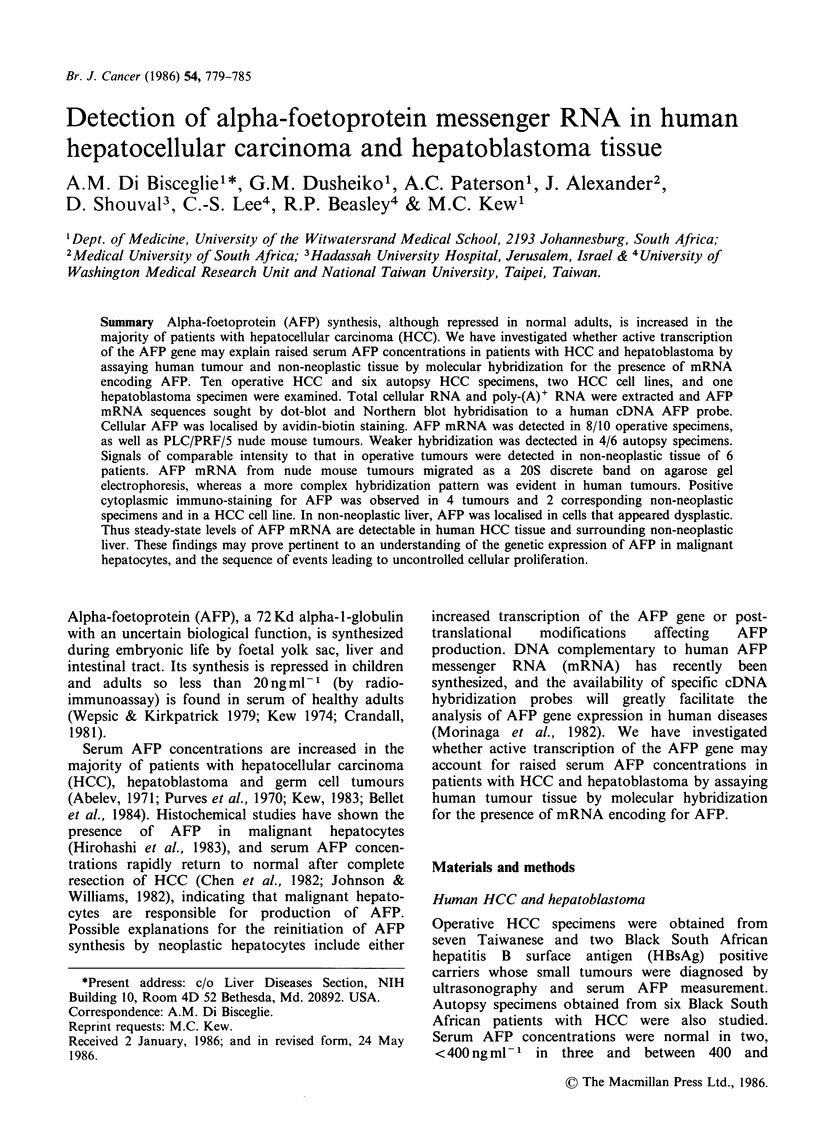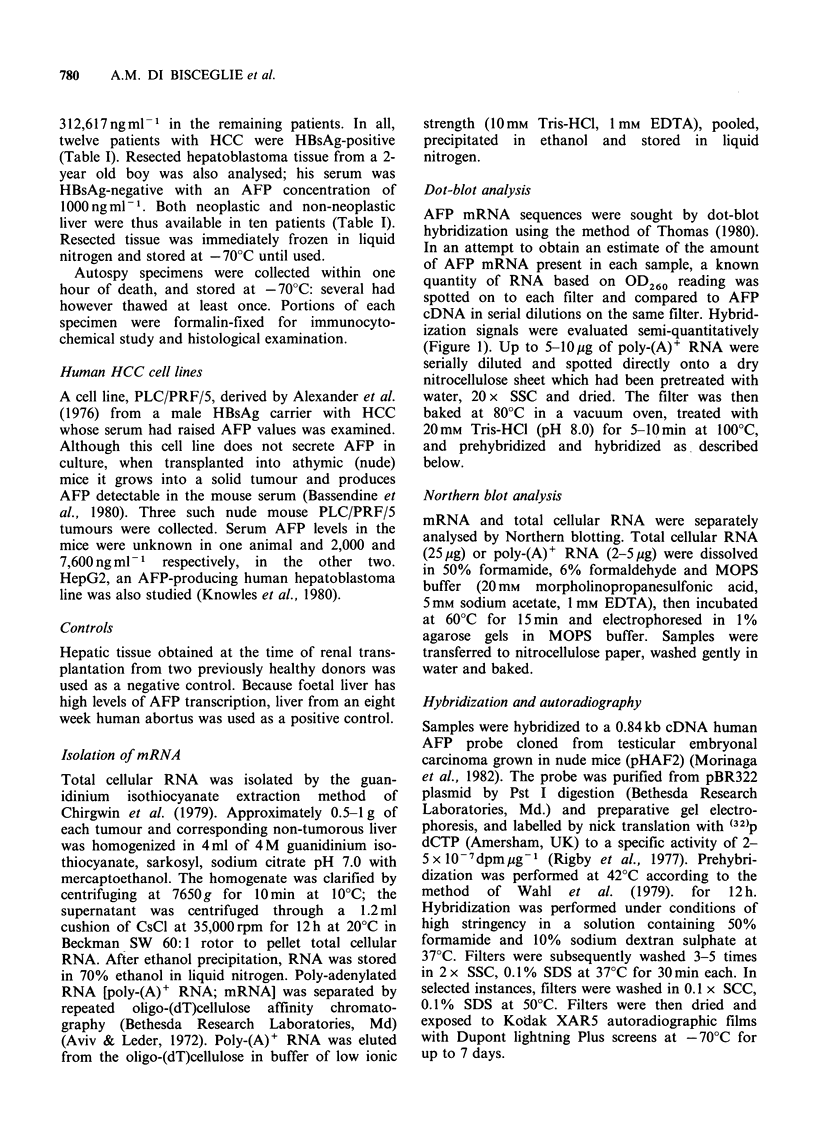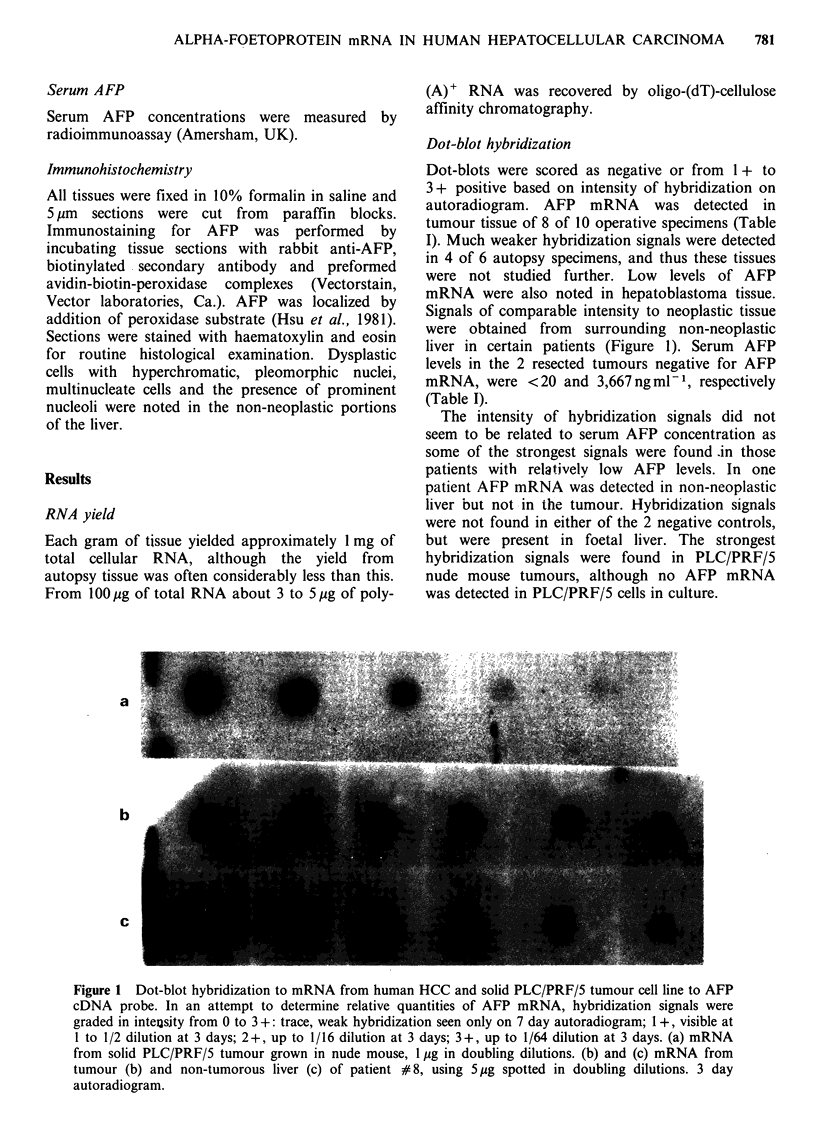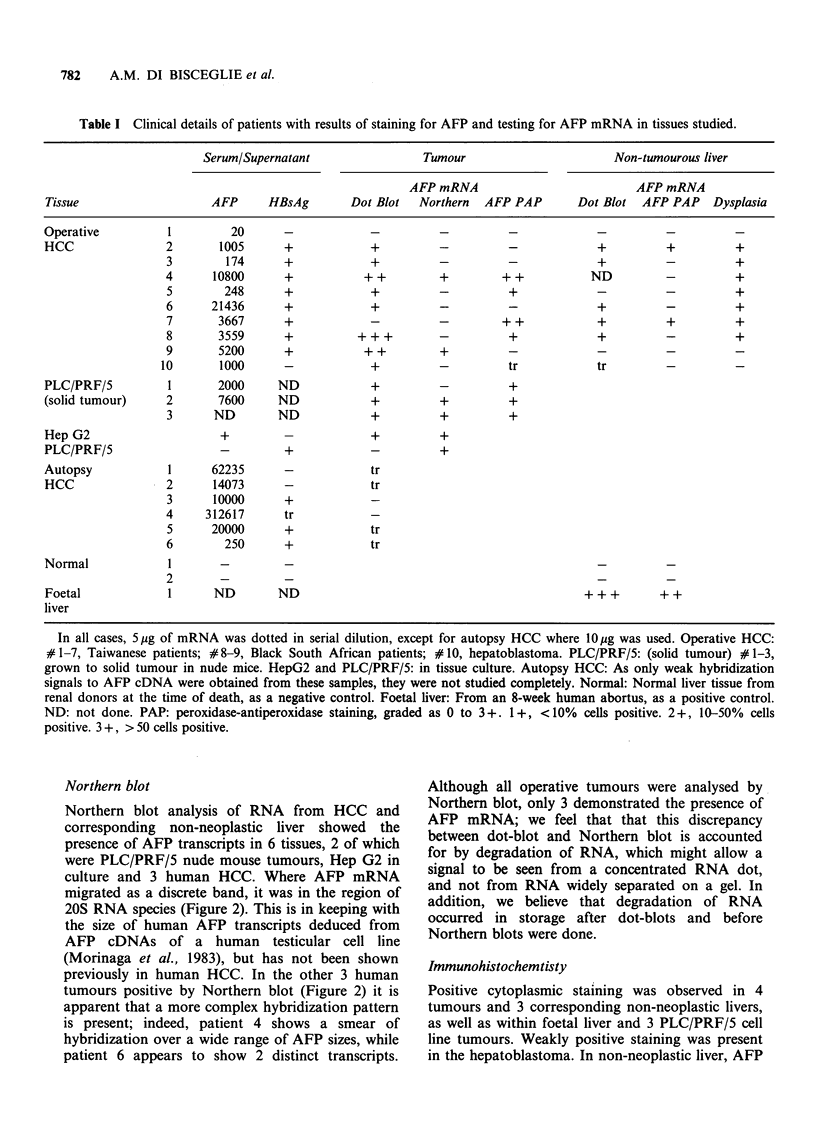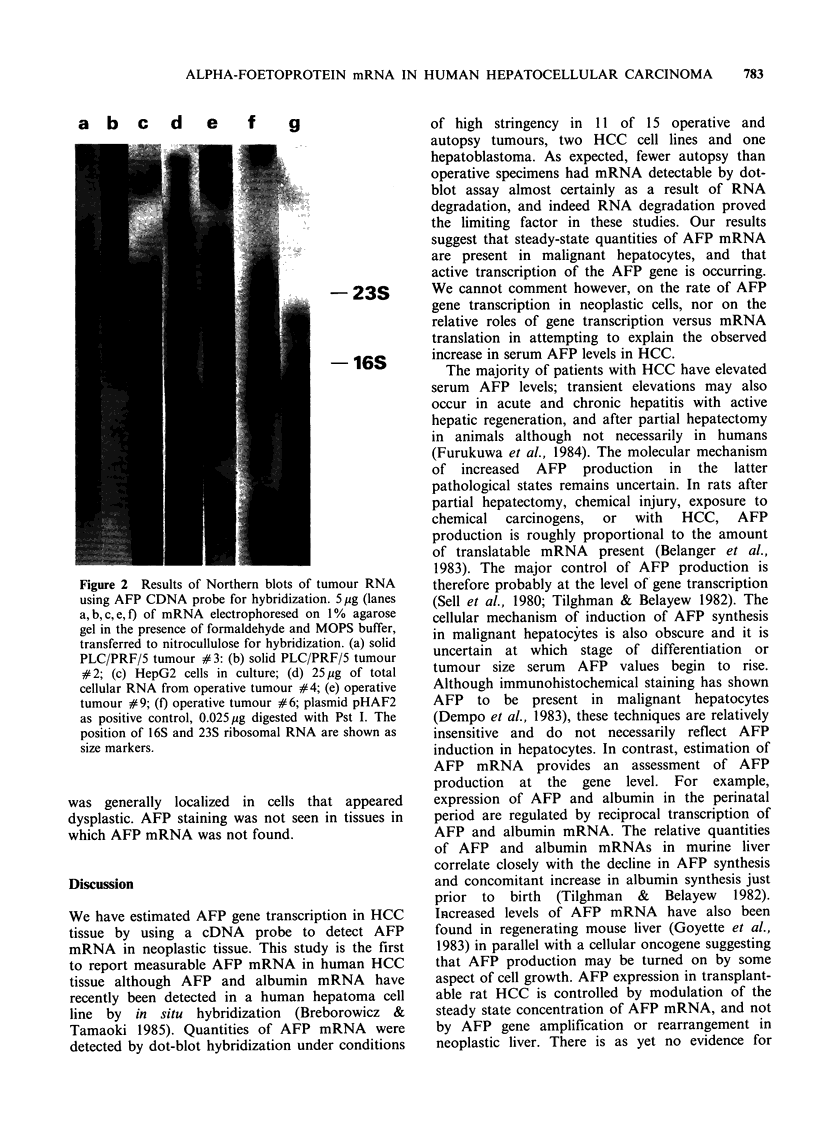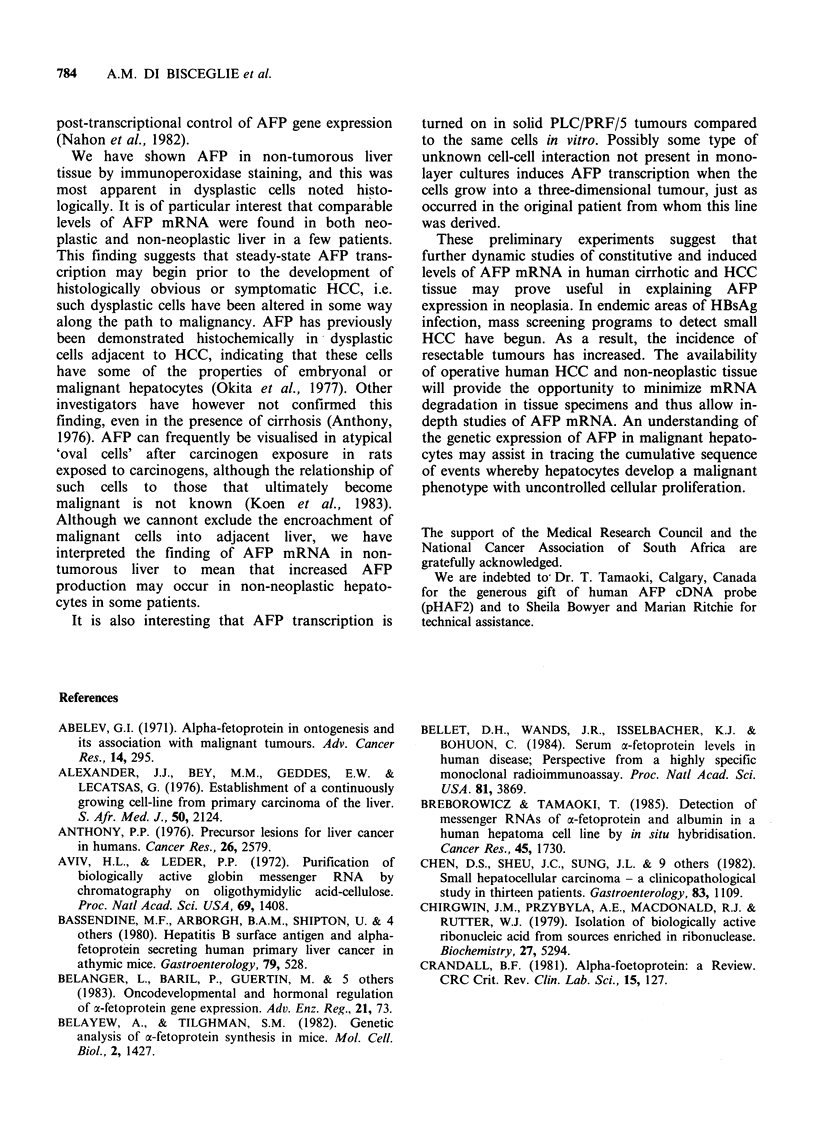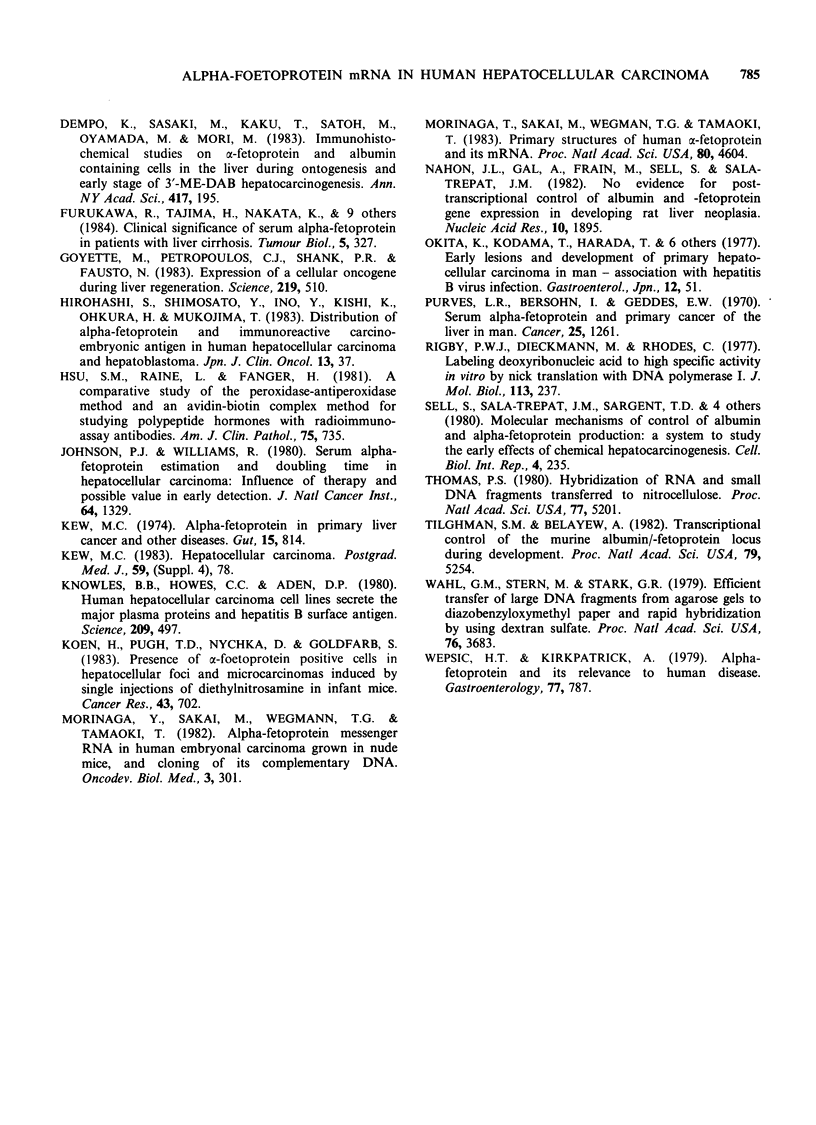# Detection of alpha-foetoprotein messenger RNA in human hepatocellular carcinoma and hepatoblastoma tissue.

**DOI:** 10.1038/bjc.1986.240

**Published:** 1986-11

**Authors:** A. M. Di Bisceglie, G. M. Dusheiko, A. C. Paterson, J. Alexander, D. Shouval, C. S. Lee, R. P. Beasley, M. C. Kew

## Abstract

**Images:**


*Br. J. Cancer* (1986) **54**, 779–785

### Detection of alpha-foetoprotein messenger RNA in human hepatocellular carcinoma and hepatoblastoma tissue

A.M. Di Bisceglie[1]*, G.M. Dusheiko[1], A.C. Paterson[1], J. Alexander[2], D. Shouval[3], C.-S. Lee[4], R.P. Beasley[4] & M.C. Kew[1]

[1]*Dept. of Medicine, University of the Witwatersrand Medical School, 2193 Johannesburg, South Africa;* [2]*Medical University of South Africa;* [3]*Hadassah University Hospital, Jerusalem, Israel &* [4]*University of Washington Medical Research Unit and National Taiwan University, Taipei, Taiwan.*

**Summary** Alpha-foetoprotein (AFP) synthesis, although repressed in normal adults, is increased in the majority of patients with hepatocellular carcinoma (HCC). We have investigated whether active transcription of the AFP gene may explain raised serum AFP concentrations in patients with HCC and hepatoblastoma by assaying human tumour and non-neoplastic tissue by molecular hybridization for the presence of mRNA encoding AFP. Ten operative HCC and six autopsy HCC specimens, two HCC cell lines, and one hepatoblastoma specimen were examined. Total cellular RNA and poly-(A)$^+$ RNA were extracted and AFP mRNA sequences sought by dot-blot and Northern blot hybridisation to a human cDNA AFP probe. Cellular AFP was localised by avidin-biotin staining. AFP mRNA was detected in 8/10 operative specimens, as well as PLC/PRF/5 nude mouse tumours. Weaker hybridization was dectected in 4/6 autopsy specimens. Signals of comparable intensity to that in operative tumours were detected in non-neoplastic tissue of 6 patients. AFP mRNA from nude mouse tumours migrated as a 20S discrete band on agarose gel electrophoresis, whereas a more complex hybridization pattern was evident in human tumours. Positive cytoplasmic immuno-staining for AFP was observed in 4 tumours and 2 corresponding non-neoplastic specimens and in a HCC cell line. In non-neoplastic liver, AFP was localised in cells that appeared dysplastic. Thus steady-state levels of AFP mRNA are detectable in human HCC tissue and surrounding non-neoplastic liver. These findings may prove pertinent to an understanding of the genetic expression of AFP in malignant hepatocytes, and the sequence of events leading to uncontrolled cellular proliferation.

Alpha-foetoprotein (AFP), a 72 Kd alpha-1-globulin with an uncertain biological function, is synthesized during embryonic life by foetal yolk sac, liver and intestinal tract. Its synthesis is repressed in children and adults so less than 20 ng ml$^{-1}$ (by radio-immunoassay) is found in serum of healthy adults (Wepsic & Kirkpatrick 1979; Kew 1974; Crandall, 1981).

Serum AFP concentrations are increased in the majority of patients with hepatocellular carcinoma (HCC), hepatoblastoma and germ cell tumours (Abelev, 1971; Purves *et al.*, 1970; Kew, 1983; Bellet *et al.*, 1984). Histochemical studies have shown the presence of AFP in malignant hepatocytes (Hirohashi *et al.*, 1983), and serum AFP concentrations rapidly return to normal after complete resection of HCC (Chen *et al.*, 1982; Johnson & Williams, 1982), indicating that malignant hepatocytes are responsible for production of AFP. Possible explanations for the reinitiation of AFP synthesis by neoplastic hepatocytes include either increased transcription of the AFP gene or post-translational modifications affecting AFP production. DNA complementary to human AFP messenger RNA (mRNA) has recently been synthesized, and the availability of specific cDNA hybridization probes will greatly facilitate the analysis of AFP gene expression in human diseases (Morinaga *et al.*, 1982). We have investigated whether active transcription of the AFP gene may account for raised serum AFP concentrations in patients with HCC and hepatoblastoma by assaying human tumour tissue by molecular hybridization for the presence of mRNA encoding for AFP.

### Materials and methods

### *Human HCC and hepatoblastoma*

Operative HCC specimens were obtained from seven Taiwanese and two Black South African hepatitis B surface antigen (HBsAg) positive carriers whose small tumours were diagnosed by ultrasonography and serum AFP measurement. Autopsy specimens obtained from six Black South African patients with HCC were also studied. Serum AFP concentrations were normal in two, <400 ng ml$^{-1}$ in three and between 400 and

*Present address: c/o Liver Diseases Section, NIH Building 10, Room 4D 52 Bethesda, Md. 20892. USA.
Correspondence: A.M. Di Bisceglie.
Reprint requests: M.C. Kew.
Received 2 January, 1986; and in revised form, 24 May 1986.

© The Macmillan Press Ltd., 1986.

312,617 ng $ml^{-1}$ in the remaining patients. In all, twelve patients with HCC were HBsAg-positive (Table I). Resected hepatoblastoma tissue from a 2-year old boy was also analysed; his serum was HBsAg-negative with an AFP concentration of 1000 ng $ml^{-1}$. Both neoplastic and non-neoplastic liver were thus available in ten patients (Table I). Resected tissue was immediately frozen in liquid nitrogen and stored at $-70°C$ until used.

Autospy specimens were collected within one hour of death, and stored at $-70°C$: several had however thawed at least once. Portions of each specimen were formalin-fixed for immunocytochemical study and histological examination.

*Human HCC cell lines*

A cell line, PLC/PRF/5, derived by Alexander *et al.* (1976) from a male HBsAg carrier with HCC whose serum had raised AFP values was examined. Although this cell line does not secrete AFP in culture, when transplanted into athymic (nude) mice it grows into a solid tumour and produces AFP detectable in the mouse serum (Bassendine *et al.,* 1980). Three such nude mouse PLC/PRF/5 tumours were collected. Serum AFP levels in the mice were unknown in one animal and 2,000 and 7,600 ng $ml^{-1}$ respectively, in the other two. HepG2, an AFP-producing human hepatoblastoma line was also studied (Knowles *et al.,* 1980).

*Controls*

Hepatic tissue obtained at the time of renal transplantation from two previously healthy donors was used as a negative control. Because foetal liver has high levels of AFP transcription, liver from an eight week human abortus was used as a positive control.

*Isolation of mRNA*

Total cellular RNA was isolated by the guanidinium isothiocyanate extraction method of Chirgwin *et al.* (1979). Approximately 0.5–1 g of each tumour and corresponding non-tumorous liver was homogenized in 4 ml of 4 M guanidinium isothiocyanate, sarkosyl, sodium citrate pH 7.0 with mercaptoethanol. The homogenate was clarified by centrifuging at 7650 *g* for 10 min at 10°C; the supernatant was centrifuged through a 1.2 ml cushion of CsCl at 35,000 rpm for 12 h at 20°C in Beckman SW 60:1 rotor to pellet total cellular RNA. After ethanol precipitation, RNA was stored in 70% ethanol in liquid nitrogen. Poly-adenylated RNA [poly-$(A)^+$ RNA; mRNA] was separated by repeated oligo-(dT)cellulose affinity chromatography (Bethesda Research Laboratories, Md) (Aviv & Leder, 1972). Poly-$(A)^+$ RNA was eluted from the oligo-(dT)cellulose in buffer of low ionic strength (10 mM Tris-HCl, 1 mM EDTA), pooled, precipitated in ethanol and stored in liquid nitrogen.

*Dot-blot analysis*

AFP mRNA sequences were sought by dot-blot hybridization using the method of Thomas (1980). In an attempt to obtain an estimate of the amount of AFP mRNA present in each sample, a known quantity of RNA based on $OD_{260}$ reading was spotted on to each filter and compared to AFP cDNA in serial dilutions on the same filter. Hybridization signals were evaluated semi-quantitatively (Figure 1). Up to 5–10 $\mu g$ of poly-$(A)^+$ RNA were serially diluted and spotted directly onto a dry nitrocellulose sheet which had been pretreated with water, 20 × SSC and dried. The filter was then baked at 80°C in a vacuum oven, treated with 20 mM Tris-HCl (pH 8.0) for 5–10 min at 100°C, and prehybridized and hybridized as described below.

*Northern blot analysis*

mRNA and total cellular RNA were separately analysed by Northern blotting. Total cellular RNA (25 $\mu g$) or poly-$(A)^+$ RNA (2–5 $\mu g$) were dissolved in 50% formamide, 6% formaldehyde and MOPS buffer (20 mM morpholinopropanesulfonic acid, 5 mM sodium acetate, 1 mM EDTA), then incubated at 60°C for 15 min and electrophoresed in 1% agarose gels in MOPS buffer. Samples were transferred to nitrocellulose paper, washed gently in water and baked.

*Hybridization and autoradiography*

Samples were hybridized to a 0.84 kb cDNA human AFP probe cloned from testicular embryonal carcinoma grown in nude mice (pHAF2) (Morinaga *et al.,* 1982). The probe was purified from pBR322 plasmid by Pst I digestion (Bethesda Research Laboratories, Md.) and preparative gel electrophoresis, and labelled by nick translation with $^{(32)}p$ dCTP (Amersham, UK) to a specific activity of 2–$5 \times 10^{-7}$ dpm $\mu g^{-1}$ (Rigby *et al.,* 1977). Prehybridization was performed at 42°C according to the method of Wahl *et al.* (1979). for 12 h. Hybridization was performed under conditions of high stringency in a solution containing 50% formamide and 10% sodium dextran sulphate at 37°C. Filters were subsequently washed 3–5 times in 2 × SSC, 0.1% SDS at 37°C for 30 min each. In selected instances, filters were washed in 0.1 × SCC, 0.1% SDS at 50°C. Filters were then dried and exposed to Kodak XAR5 autoradiographic films with Dupont lightning Plus screens at $-70°C$ for up to 7 days.

*Serum AFP*

Serum AFP concentrations were measured by radioimmunoassay (Amersham, UK).

*Immunohistochemistry*

All tissues were fixed in 10% formalin in saline and 5 $\mu$m sections were cut from paraffin blocks. Immunostaining for AFP was performed by incubating tissue sections with rabbit anti-AFP, biotinylated secondary antibody and preformed avidin-biotin-peroxidase complexes (Vectorstain, Vector laboratories, Ca.). AFP was localized by addition of peroxidase substrate (Hsu *et al.*, 1981). Sections were stained with haematoxylin and eosin for routine histological examination. Dysplastic cells with hyperchromatic, pleomorphic nuclei, multinucleate cells and the presence of prominent nucleoli were noted in the non-neoplastic portions of the liver.

### Results

*RNA yield*

Each gram of tissue yielded approximately 1 mg of total cellular RNA, although the yield from autopsy tissue was often considerably less than this. From 100 $\mu$g of total RNA about 3 to 5 $\mu$g of poly-(A)$^+$ RNA was recovered by oligo-(dT)-cellulose affinity chromatography.

*Dot-blot hybridization*

Dot-blots were scored as negative or from 1+ to 3+ positive based on intensity of hybridization on autoradiogram. AFP mRNA was detected in tumour tissue of 8 of 10 operative specimens (Table I). Much weaker hybridization signals were detected in 4 of 6 autopsy specimens, and thus these tissues were not studied further. Low levels of AFP mRNA were also noted in hepatoblastoma tissue. Signals of comparable intensity to neoplastic tissue were obtained from surrounding non-neoplastic liver in certain patients (Figure 1). Serum AFP levels in the 2 resected tumours negative for AFP mRNA, were <20 and 3,667 ng ml$^{-1}$, respectively (Table I).

The intensity of hybridization signals did not seem to be related to serum AFP concentration as some of the strongest signals were found in those patients with relatively low AFP levels. In one patient AFP mRNA was detected in non-neoplastic liver but not in the tumour. Hybridization signals were not found in either of the 2 negative controls, but were present in foetal liver. The strongest hybridization signals were found in PLC/PRF/5 nude mouse tumours, although no AFP mRNA was detected in PLC/PRF/5 cells in culture.

**Figure 1** Dot-blot hybridization to mRNA from human HCC and solid PLC/PRF/5 tumour cell line to AFP cDNA probe. In an attempt to determine relative quantities of AFP mRNA, hybridization signals were graded in intensity from 0 to 3+: trace, weak hybridization seen only on 7 day autoradiogram; 1+, visible at 1 to 1/2 dilution at 3 days; 2+, up to 1/16 dilution at 3 days; 3+, up to 1/64 dilution at 3 days. (a) mRNA from solid PLC/PRF/5 tumour grown in nude mouse, 1 $\mu$g in doubling dilutions. (b) and (c) mRNA from tumour (b) and non-tumorous liver (c) of patient #8, using 5 $\mu$g spotted in doubling dilutions. 3 day autoradiogram.

**Table I** Clinical details of patients with results of staining for AFP and testing for AFP mRNA in tissues studied.

| | | *Serum/Supernatant* | | *Tumour* | | | *Non-tumourous liver* | | |
|---|---|---|---|---|---|---|---|---|---|
| | | | | *AFP mRNA* | | | *AFP mRNA* | | |
| *Tissue* | | *AFP* | *HBsAg* | *Dot Blot* | *Northern* | *AFP PAP* | *Dot Blot* | *AFP PAP* | *Dysplasia* |
| Operative | 1 | 20 | – | – | – | – | – | – | – |
| HCC | 2 | 1005 | + | + | – | – | + | + | + |
| | 3 | 174 | + | + | – | – | + | – | + |
| | 4 | 10800 | + | ++ | + | ++ | ND | – | + |
| | 5 | 248 | + | + | – | + | – | – | + |
| | 6 | 21436 | + | + | – | – | + | – | + |
| | 7 | 3667 | + | – | – | ++ | + | + | + |
| | 8 | 3559 | + | +++ | – | + | + | – | + |
| | 9 | 5200 | + | ++ | + | – | – | – | – |
| | 10 | 1000 | – | + | – | tr | tr | – | – |
| PLC/PRF/5 | 1 | 2000 | ND | + | – | + | | | |
| (solid tumour) | 2 | 7600 | ND | + | + | + | | | |
| | 3 | ND | ND | + | + | + | | | |
| Hep G2 | | + | – | + | + | | | | |
| PLC/PRF/5 | | – | + | – | + | | | | |
| Autopsy | 1 | 62235 | – | tr | | | | | |
| HCC | 2 | 14073 | – | tr | | | | | |
| | 3 | 10000 | + | – | | | | | |
| | 4 | 312617 | tr | – | | | | | |
| | 5 | 20000 | + | tr | | | | | |
| | 6 | 250 | + | tr | | | | | |
| Normal | 1 | – | – | | | | – | – | |
| | 2 | – | – | | | | – | – | |
| Foetal liver | 1 | ND | ND | | | | +++ | ++ | |

In all cases, 5 μg of mRNA was dotted in serial dilution, except for autopsy HCC where 10 μg was used. Operative HCC: #1–7, Taiwanese patients; #8–9, Black South African patients; #10, hepatoblastoma. PLC/PRF/5: (solid tumour) #1–3, grown to solid tumour in nude mice. HepG2 and PLC/PRF/5: in tissue culture. Autopsy HCC: As only weak hybridization signals to AFP cDNA were obtained from these samples, they were not studied completely. Normal: Normal liver tissue from renal donors at the time of death, as a negative control. Foetal liver: From an 8-week human abortus, as a positive control. ND: not done. PAP: peroxidase-antiperoxidase staining, graded as 0 to 3+. 1+, <10% cells positive. 2+, 10–50% cells positive. 3+, >50 cells positive.

*Northern blot*

Northern blot analysis of RNA from HCC and corresponding non-neoplastic liver showed the presence of AFP transcripts in 6 tissues, 2 of which were PLC/PRF/5 nude mouse tumours, Hep G2 in culture and 3 human HCC. Where AFP mRNA migrated as a discrete band, it was in the region of 20S RNA species (Figure 2). This is in keeping with the size of human AFP transcripts deduced from AFP cDNAs of a human testicular cell line (Morinaga *et al.*, 1983), but has not been shown previously in human HCC. In the other 3 human tumours positive by Northern blot (Figure 2) it is apparent that a more complex hybridization pattern is present; indeed, patient 4 shows a smear of hybridization over a wide range of AFP sizes, while patient 6 appears to show 2 distinct transcripts. Although all operative tumours were analysed by Northern blot, only 3 demonstrated the presence of AFP mRNA; we feel that that this discrepancy between dot-blot and Northern blot is accounted for by degradation of RNA, which might allow a signal to be seen from a concentrated RNA dot, and not from RNA widely separated on a gel. In addition, we believe that degradation of RNA occurred in storage after dot-blots and before Northern blots were done.

*Immunohistochemtisty*

Positive cytoplasmic staining was observed in 4 tumours and 3 corresponding non-neoplastic livers, as well as within foetal liver and 3 PLC/PRF/5 cell line tumours. Weakly positive staining was present in the hepatoblastoma. In non-neoplastic liver, AFP

**Figure 2** Results of Northern blots of tumour RNA using AFP CDNA probe for hybridization. 5 $\mu$g (lanes a, b, c, e, f) of mRNA electrophoresed on 1% agarose gel in the presence of formaldehyde and MOPS buffer, transferred to nitroculllulose for hybridization. (a) solid PLC/PRF/5 tumour #3: (b) solid PLC/PRF/5 tumour #2; (c) HepG2 cells in culture; (d) 25 $\mu$g of total cellular RNA from operative tumour #4; (e) operative tumour #9; (f) operative tumour #6; plasmid pHAF2 as positive control, 0.025 $\mu$g digested with Pst I. The position of 16S and 23S ribosomal RNA are shown as size markers.

was generally localized in cells that appeared dysplastic. AFP staining was not seen in tissues in which AFP mRNA was not found.

### Discussion

We have estimated AFP gene transcription in HCC tissue by using a cDNA probe to detect AFP mRNA in neoplastic tissue. This study is the first to report measurable AFP mRNA in human HCC tissue although AFP and albumin mRNA have recently been detected in a human hepatoma cell line by *in situ* hybridization (Breborowicz & Tamaoki 1985). Quantities of AFP mRNA were detected by dot-blot hybridization under conditions of high stringency in 11 of 15 operative and autopsy tumours, two HCC cell lines and one hepatoblastoma. As expected, fewer autopsy than operative specimens had mRNA detectable by dot-blot assay almost certainly as a result of RNA degradation, and indeed RNA degradation proved the limiting factor in these studies. Our results suggest that steady-state quantities of AFP mRNA are present in malignant hepatocytes, and that active transcription of the AFP gene is occurring. We cannot comment however, on the rate of AFP gene transcription in neoplastic cells, nor on the relative roles of gene transcription versus mRNA translation in attempting to explain the observed increase in serum AFP levels in HCC.

The majority of patients with HCC have elevated serum AFP levels; transient elevations may also occur in acute and chronic hepatitis with active hepatic regeneration, and after partial hepatectomy in animals although not necessarily in humans (Furukuwa *et al.*, 1984). The molecular mechanism of increased AFP production in the latter pathological states remains uncertain. In rats after partial hepatectomy, chemical injury, exposure to chemical carcinogens, or with HCC, AFP production is roughly proportional to the amount of translatable mRNA present (Belanger *et al.*, 1983). The major control of AFP production is therefore probably at the level of gene transcription (Sell *et al.*, 1980; Tilghman & Belayew 1982). The cellular mechanism of induction of AFP synthesis in malignant hepatocytes is also obscure and it is uncertain at which stage of differentiation or tumour size serum AFP values begin to rise. Although immunohistochemical staining has shown AFP to be present in malignant hepatocytes (Dempo *et al.*, 1983), these techniques are relatively insensitive and do not necessarily reflect AFP induction in hepatocytes. In contrast, estimation of AFP mRNA provides an assessment of AFP production at the gene level. For example, expression of AFP and albumin in the perinatal period are regulated by reciprocal transcription of AFP and albumin mRNA. The relative quantities of AFP and albumin mRNAs in murine liver correlate closely with the decline in AFP synthesis and concomitant increase in albumin synthesis just prior to birth (Tilghman & Belayew 1982). Increased levels of AFP mRNA have also been found in regenerating mouse liver (Goyette *et al.*, 1983) in parallel with a cellular oncogene suggesting that AFP production may be turned on by some aspect of cell growth. AFP expression in transplantable rat HCC is controlled by modulation of the steady state concentration of AFP mRNA, and not by AFP gene amplification or rearrangement in neoplastic liver. There is as yet no evidence for post-transcriptional control of AFP gene expression (Nahon *et al.*, 1982).

We have shown AFP in non-tumorous liver tissue by immunoperoxidase staining, and this was most apparent in dysplastic cells noted histologically. It is of particular interest that comparable levels of AFP mRNA were found in both neoplastic and non-neoplastic liver in a few patients. This finding suggests that steady-state AFP transcription may begin prior to the development of histologically obvious or symptomatic HCC, i.e. such dysplastic cells have been altered in some way along the path to malignancy. AFP has previously been demonstrated histochemically in dysplastic cells adjacent to HCC, indicating that these cells have some of the properties of embryonal or malignant hepatocytes (Okita *et al.*, 1977). Other investigators have however not confirmed this finding, even in the presence of cirrhosis (Anthony, 1976). AFP can frequently be visualised in atypical 'oval cells' after carcinogen exposure in rats exposed to carcinogens, although the relationship of such cells to those that ultimately become malignant is not known (Koen *et al.*, 1983). Although we cannont exclude the encroachment of malignant cells into adjacent liver, we have interpreted the finding of AFP mRNA in non-tumorous liver to mean that increased AFP production may occur in non-neoplastic hepatocytes in some patients.

It is also interesting that AFP transcription is turned on in solid PLC/PRF/5 tumours compared to the same cells *in vitro*. Possibly some type of unknown cell-cell interaction not present in monolayer cultures induces AFP transcription when the cells grow into a three-dimensional tumour, just as occurred in the original patient from whom this line was derived.

These preliminary experiments suggest that further dynamic studies of constitutive and induced levels of AFP mRNA in human cirrhotic and HCC tissue may prove useful in explaining AFP expression in neoplasia. In endemic areas of HBsAg infection, mass screening programs to detect small HCC have begun. As a result, the incidence of resectable tumours has increased. The availability of operative human HCC and non-neoplastic tissue will provide the opportunity to minimize mRNA degradation in tissue specimens and thus allow in-depth studies of AFP mRNA. An understanding of the genetic expression of AFP in malignant hepatocytes may assist in tracing the cumulative sequence of events whereby hepatocytes develop a malignant phenotype with uncontrolled cellular proliferation.

The support of the Medical Research Council and the National Cancer Association of South Africa are gratefully acknowledged.

We are indebted to Dr. T. Tamaoki, Calgary, Canada for the generous gift of human AFP cDNA probe (pHAF2) and to Sheila Bowyer and Marian Ritchie for technical assistance.

### References

ABELEV, G.I. (1971). Alpha-fetoprotein in ontogenesis and its association with malignant tumours. *Adv. Cancer Res.*, **14,** 295.

ALEXANDER, J.J., BEY, M.M., GEDDES, E.W. & LECATSAS, G. (1976). Establishment of a continuously growing cell-line from primary carcinoma of the liver. *S. Afr. Med. J.*, **50,** 2124.

ANTHONY, P.P. (1976). Precursor lesions for liver cancer in humans. *Cancer Res.*, **26,** 2579.

AVIV, H.L., & LEDER, P.P. (1972). Purification of biologically active globin messenger RNA by chromatography on oligothymidylic acid-cellulose. *Proc. Natl Acad. Sci. USA*, **69,** 1408.

BASSENDINE, M.F., ARBORGH, B.A.M., SHIPTON, U. & 4 others (1980). Hepatitis B surface antigen and alpha-fetoprotein secreting human primary liver cancer in athymic mice. *Gastroenterology*, **79,** 528.

BELANGER, L., BARIL, P., GUERTIN, M. & 5 others (1983). Oncodevelopmental and hormonal regulation of α-fetoprotein gene expression. *Adv. Enz. Reg.*, **21,** 73.

BELAYEW, A., & TILGHMAN, S.M. (1982). Genetic analysis of α-fetoprotein synthesis in mice. *Mol. Cell. Biol.*, **2,** 1427.

BELLET, D.H., WANDS, J.R., ISSELBACHER, K.J. & BOHUON, C. (1984). Serum α-fetoprotein levels in human disease; Perspective from a highly specific monoclonal radioimmunoassay. *Proc. Natl Acad. Sci. USA*. **81,** 3869.

BREBOROWICZ & TAMAOKI, T. (1985). Detection of messenger RNAs of α-fetoprotein and albumin in a human hepatoma cell line by *in situ* hybridisation. *Cancer Res.*, **45,** 1730.

CHEN, D.S., SHEU, J.C., SUNG, J.L. & 9 others (1982). Small hepatocellular carcinoma – a clinicopathological study in thirteen patients. *Gastroenterology*, **83,** 1109.

CHIRGWIN, J.M., PRZYBYLA, A.E., MACDONALD, R.J. & RUTTER, W.J. (1979). Isolation of biologically active ribonucleic acid from sources enriched in ribonuclease. *Biochemistry*, **27,** 5294.

CRANDALL, B.F. (1981). Alpha-foetoprotein: a Review. CRC Crit. Rev. *Clin. Lab. Sci.*, **15,** 127.

DEMPO, K., SASAKI, M., KAKU, T., SATOH, M., OYAMADA, M. & MORI, M. (1983). Immunohistochemical studies on α-fetoprotein and albumin containing cells in the liver during ontogenesis and early stage of 3′-ME-DAB hepatocarcinogenesis. *Ann. NY Acad. Sci.*, **417**, 195.

FURUKAWA, R., TAJIMA, H., NAKATA, K., & 9 others (1984). Clinical significance of serum alpha-fetoprotein in patients with liver cirrhosis. *Tumour Biol.*, **5**, 327.

GOYETTE, M., PETROPOULOS, C.J., SHANK, P.R. & FAUSTO, N. (1983). Expression of a cellular oncogene during liver regeneration. *Science*, **219**, 510.

HIROHASHI, S., SHIMOSATO, Y., INO, Y., KISHI, K., OHKURA, H. & MUKOJIMA, T. (1983). Distribution of alpha-fetoprotein and immunoreactive carcinoembryonic antigen in human hepatocellular carcinoma and hepatoblastoma. *Jpn. J. Clin. Oncol.* **13**, 37.

HSU, S.M., RAINE, L. & FANGER, H. (1981). A comparative study of the peroxidase-antiperoxidase method and an avidin-biotin complex method for studying polypeptide hormones with radioimmunoassay antibodies. *Am. J. Clin. Pathol.*, **75**, 735.

JOHNSON, P.J. & WILLIAMS, R. (1980). Serum alpha-fetoprotein estimation and doubling time in hepatocellular carcinoma: Influence of therapy and possible value in early detection. *J. Natl Cancer Inst.*, **64**, 1329.

KEW, M.C. (1974). Alpha-fetoprotein in primary liver cancer and other diseases. *Gut*, **15**, 814.

KEW, M.C. (1983). Hepatocellular carcinoma. *Postgrad. Med. J.*, **59**, (Suppl. 4), 78.

KNOWLES, B.B., HOWES, C.C. & ADEN, D.P. (1980). Human hepatocellular carcinoma cell lines secrete the major plasma proteins and hepatitis B surface antigen. *Science*, **209**, 497.

KOEN, H., PUGH, T.D., NYCHKA, D. & GOLDFARB, S. (1983). Presence of α-foetoprotein positive cells in hepatocellular foci and microcarcinomas induced by single injections of diethylnitrosamine in infant mice. *Cancer Res.*, **43**, 702.

MORINAGA, Y., SAKAI, M., WEGMANN, T.G. & TAMAOKI, T. (1982). Alpha-fetoprotein messenger RNA in human embryonal carcinoma grown in nude mice, and cloning of its complementary DNA. *Oncodev. Biol. Med.*, **3**, 301.

MORINAGA, T., SAKAI, M., WEGMAN, T.G. & TAMAOKI, T. (1983). Primary structures of human α-fetoprotein and its mRNA. *Proc. Natl Acad. Sci. USA*, **80**, 4604.

NAHON, J.L., GAL, A., FRAIN, M., SELL, S. & SALA-TREPAT, J.M. (1982). No evidence for post-transcriptional control of albumin and -fetoprotein gene expression in developing rat liver neoplasia. *Nucleic Acid Res.*, **10**, 1895.

OKITA, K., KODAMA, T., HARADA, T. & 6 others (1977). Early lesions and development of primary hepatocellular carcinoma in man – association with hepatitis B virus infection. *Gastroenterol., Jpn.*, **12**, 51.

PURVES, L.R., BERSOHN, I. & GEDDES, E.W. (1970). Serum alpha-fetoprotein and primary cancer of the liver in man. *Cancer*, **25**, 1261.

RIGBY, P.W.J., DIECKMANN, M. & RHODES, C. (1977). Labeling deoxyribonucleic acid to high specific activity *in vitro* by nick translation with DNA polymerase I. *J. Mol. Biol.*, **113**, 237.

SELL, S., SALA-TREPAT, J.M., SARGENT, T.D. & 4 others (1980). Molecular mechanisms of control of albumin and alpha-fetoprotein production: a system to study the early effects of chemical hepatocarcinogenesis. *Cell. Biol. Int. Rep.*, **4**, 235.

THOMAS, P.S. (1980). Hybridization of RNA and small DNA fragments transferred to nitrocellulose. *Proc. Natl Acad. Sci. USA*, **77**, 5201.

TILGHMAN, S.M. & BELAYEW, A. (1982). Transcriptional control of the murine albumin/-fetoprotein locus during development. *Proc. Natl Acad. Sci. USA*, **79**, 5254.

WAHL, G.M., STERN, M. & STARK, G.R. (1979). Efficient transfer of large DNA fragments from agarose gels to diazobenzyloxymethyl paper and rapid hybridization by using dextran sulfate. *Proc. Natl Acad. Sci. USA*, **76**, 3683.

WEPSIC, H.T. & KIRKPATRICK, A. (1979). Alpha-fetoprotein and its relevance to human disease. *Gastroenterology*, **77**, 787.